# Supplementary material for: Resolving the full spectrum of human genome variation using Linked-Reads
Source: Genome Res. 2019 Apr;29(4):635–45. doi: 10.1101/gr.234443.118 (PMC6442396; doi:10.1101/gr.234443.118)
Supplement: Supplemental Material [file supp_gr.234443.118_Supplemental_longranger-2.2.zip › longranger-2.2.tar.bz2!/lr2.2-public/lib/stan/cmdstan/stan/doc/api/html/structstan_1_1math_1_1coupled__ode__observer.html]

Stan: stan::math::coupled\_ode\_observer Struct Reference


|  |  |
| --- | --- |
|  | Stan  2.5.0  probability, sampling & optimization |


- Main Page
- Related Pages
- Namespaces
- Classes
- Files

- Class List
- Class Index
- Class Hierarchy
- Class Members

All Classes Namespaces Files Functions Variables Typedefs Enumerations Enumerator Friends Macros Pages

- stan
- math
- coupled\_ode\_observer

Public Member Functions |
Public Attributes |
List of all members

stan::math::coupled\_ode\_observer Struct Reference

Observer for the coupled states.
More...

`#include <coupled_ode_observer.hpp>`

|  |  |
| --- | --- |
| Public Member Functions | |
|  | coupled\_ode\_observer (std::vector< std::vector< double > > &y\_coupled) |
|  | Construct a coupled ODE observer from the specified coupled vector. More... |
|  | |
| void | operator() (const std::vector< double > &coupled\_state, const double t) |
|  | Callback function for Boost's ODE solver to record values. More... |
|  | |

|  |  |
| --- | --- |
| Public Attributes | |
| std::vector< std::vector  < double > > & | y\_coupled\_ |
|  | |
| int | n\_ |
|  | |

## Detailed Description

Observer for the coupled states.

Holds a reference to an externally defined vector of vectors passed in at construction time.

Definition at line 15 of file coupled\_ode\_observer.hpp.

## Constructor & Destructor Documentation

|  |  |  |  |  |  |
| --- | --- | --- | --- | --- | --- |
| stan::math::coupled\_ode\_observer::coupled\_ode\_observer | ( | std::vector< std::vector< double > > & | *y\_coupled* | ) |  |

inline

Construct a coupled ODE observer from the specified coupled vector.

Parameters
:   |  |  |
    | --- | --- |
    | y\_coupled | reference to a vector of vector of doubles. |

Definition at line 26 of file coupled\_ode\_observer.hpp.

## Member Function Documentation

|  |  |  |  |
| --- | --- | --- | --- |
| void stan::math::coupled\_ode\_observer::operator() | ( | const std::vector< double > & | *coupled\_state*, |
|  |  | const double | *t* |
|  | ) |  |  |

inline

Callback function for Boost's ODE solver to record values.

Parameters
:   |  |  |
    | --- | --- |
    | coupled\_state | solution at the specified time. |
    | t | time of solution. |

Definition at line 36 of file coupled\_ode\_observer.hpp.

## Member Data Documentation

int stan::math::coupled\_ode\_observer::n\_

Definition at line 18 of file coupled\_ode\_observer.hpp.

std::vector<std::vector<double> >& stan::math::coupled\_ode\_observer::y\_coupled\_

Definition at line 17 of file coupled\_ode\_observer.hpp.

---

The documentation for this struct was generated from the following file:

- src/stan/math/ode/coupled\_ode\_observer.hpp


---

[ Stan Home Page ]

*© 2011–2014,
Stan Development Team.*
